# Supplementary material for: COZOID: contact zone identifier for visual analysis of protein-protein interactions
Source: BMC Bioinformatics. 2018 Apr 6;19:125. doi: 10.1186/s12859-018-2113-6 (PMC5889581; doi:10.1186/s12859-018-2113-6)
Supplement: Supplementary file 2 — Software build. Executable binary file of the software tool. (ZIP 118,784 kb) [file 12859_2018_2113_MOESM2_ESM.zip › cozoid/java/docs/beansbinding-1.2.1-doc.zip!/org/jdesktop/el/impl/lang/FunctionMapperImpl.Function.html]

FunctionMapperImpl.Function (Beans Binding)


---


|  |  |  |
| --- | --- | --- |
| **Overview** \| **Package** \| **Class** \| **Use** \| **Tree** \| **Deprecated** \| **Index** \| **Help** | |  |
| **PREV CLASS**   **NEXT CLASS** | **FRAMES**    **NO FRAMES**     **All Classes** |  |
| SUMMARY: NESTED \| FIELD \| CONSTR \| METHOD | DETAIL: FIELD \| CONSTR \| METHOD |  |


---


## org.jdesktop.el.impl.lang Class FunctionMapperImpl.Function

```
java.lang.Object
  org.jdesktop.el.impl.lang.FunctionMapperImpl.Function
```

**All Implemented Interfaces:**: java.io.Externalizable, java.io.Serializable

**Enclosing class:**: FunctionMapperImpl

---

``` public static class FunctionMapperImpl.Function extends java.lang.Object implements java.io.Externalizable ```

**See Also:**: Serialized Form

---

| **Field Summary** | |
| --- | --- |
| `protected  java.lang.String` | `localName` |
| `protected  java.lang.reflect.Method` | `m` |
| `protected  java.lang.String` | `name` |
| `protected  java.lang.String` | `owner` |
| `protected  java.lang.String` | `prefix` |
| `protected  java.lang.String[]` | `types` |


**Constructor Summary**

`FunctionMapperImpl.Function()`

`FunctionMapperImpl.Function(java.lang.String prefix,
java.lang.String localName,
java.lang.reflect.Method m)`


| **Method Summary** | |
| --- | --- |
| `boolean` | `equals(java.lang.Object obj)` |
| `java.lang.reflect.Method` | `getMethod()` |
| `int` | `hashCode()` |
| `boolean` | `matches(java.lang.String prefix, java.lang.String localName)` |
| `void` | `readExternal(java.io.ObjectInput in)` |
| `void` | `writeExternal(java.io.ObjectOutput out)` |

**Methods inherited from class java.lang.Object**

`clone, finalize, getClass, notify, notifyAll, toString, wait, wait, wait`

**Field Detail**

### m

```
protected transient java.lang.reflect.Method m
```

---


### owner

```
protected java.lang.String owner
```

---


### name

```
protected java.lang.String name
```

---


### types

```
protected java.lang.String[] types
```

---


### prefix

```
protected java.lang.String prefix
```

---


### localName

```
protected java.lang.String localName
```


**Constructor Detail**

### FunctionMapperImpl.Function

```
public FunctionMapperImpl.Function(java.lang.String prefix,
                                   java.lang.String localName,
                                   java.lang.reflect.Method m)
```

---


### FunctionMapperImpl.Function

```
public FunctionMapperImpl.Function()
```


**Method Detail**

### writeExternal

```
public void writeExternal(java.io.ObjectOutput out)
                   throws java.io.IOException
```

:   **Specified by:**: `writeExternal` in interface `java.io.Externalizable`
:   **Throws:**: `java.io.IOException`

---


### readExternal

```
public void readExternal(java.io.ObjectInput in)
                  throws java.io.IOException,
                         java.lang.ClassNotFoundException
```

:   **Specified by:**: `readExternal` in interface `java.io.Externalizable`
:   **Throws:**: `java.io.IOException`: `java.lang.ClassNotFoundException`

---


### getMethod

```
public java.lang.reflect.Method getMethod()
```

---


### matches

```
public boolean matches(java.lang.String prefix,
                       java.lang.String localName)
```

---


### equals

```
public boolean equals(java.lang.Object obj)
```

:   **Overrides:**: `equals` in class `java.lang.Object`

---


### hashCode

```
public int hashCode()
```

:   **Overrides:**: `hashCode` in class `java.lang.Object`


---


|  |  |  |
| --- | --- | --- |
| **Overview** \| **Package** \| **Class** \| **Use** \| **Tree** \| **Deprecated** \| **Index** \| **Help** | |  |
| **PREV CLASS**   **NEXT CLASS** | **FRAMES**    **NO FRAMES**     **All Classes** |  |
| SUMMARY: NESTED \| FIELD \| CONSTR \| METHOD | DETAIL: FIELD \| CONSTR \| METHOD |  |


---
